# Distribution and molecular identification of ixodid ticks infesting cattle in Kilombero and Iringa Districts, Tanzania

**DOI:** 10.1186/s12917-023-03652-x

**Published:** 2023-08-12

**Authors:** Walter S Magesa, Isihaka Haji, Edson Kinimi, Jahashi S Nzalawahe, Rudovick Kazwala

**Affiliations:** 1 Department of Veterinary Microbiology, Parasitology and Biotechnology, Sokoine University of Agriculture, P.O. Box 3019, Morogoro, Tanzania; 2 Department of Veterinary Physiology, Biochemistry and Pharmacology, Sokoine University of Agriculture, P.O. Box 3017, Morogoro, Tanzania; 3 Department of Veterinary Medicine and Public Health, Sokoine University of Agriculture, P.O. Box 3021, Morogoro, Tanzania

**Keywords:** Ticks, Distribution, Prevalence, Burden, Cattle

## Abstract

**Background:**

Hard ticks infesting cattle are vectors of Tick-borne diseases that causes major public health problems and considerable socioeconomic losses to the livestock industry in tropical and subtropical countries. A repeated cross-sectional study was carried out by collecting ticks on cattle during the wet and dry seasons from January to August 2021 in order to determine hard tick prevalence, distribution, and abundance on cattle in Kilombero and Iringa Districts of Tanzania. The collected ticks were identified morphologically using published morphological keys under a stereomicroscope and confirmed by polymerase chain reaction (PCR) and sequencing.

**Results:**

Out of 740 examined cattle, 304, (41.08%) were infested with ticks. In total, 1,780 ticks were counted on one side of the animal’s body and doubled, whereby resulting in a total of 3,560 ticks were recorded. Individual tick burden ranged from n = 2 to 82 ticks per animal, with a mean tick burden of n = 11.7 ± 0.68 per animal. A total of 1,889 ticks were collected from infected cattle including 109 additional ticks observed while collecting ticks based on the animal’s posture when restrained to the ground. Out of 1,889 ticks, nine species from three genera were identified morphologically: 1,377 fit in the genus *Rhipicephalus*, 459 to the genus of *Amblyomma*, and 53 to the genus *Hyalomma*. *Rhipicephalus microplus *was the most prevalent (n = 909, 48.1%), followed by *Rhipicephalus evertsi *(n = 310, 16.4%), *Amblyomma lepidum *(n = 310, 16.4%), *Rhipicephalus appendiculatus* (n = 140, 7.4%), *Amblyomma gemma* (n = 120, 6.4%), *Hyalomma rufipes* (n = 50, 2.6%), *Amblyomma variegatum *(n = 29, 1.5%), *Rhipicephalus decoloratus* (n = 18, 1.0%), while the least common was *Hyalomma albiparmatum* (n = 3, 0.2%). Tick diversity was higher in Iringa compared to Kilombero District. Tick prevalence was higher in wet season (n = 148, 41.11%). Ticks were widely distributed in different parts of the host body, with the highest distribution on zone 4 (n = 1,060, 56.11%), which includes groin, flank, abdomen, and around inner thigh of the hind legs and the lowest distribution on zone 2 (n = 14, 0.74%), which includes the back surface of the body. Both *Amblyomma lepidum* and *Rhipicephalus microplus* species were found in all the five body zones, and with the highest proportions recorded on zone 4: *A. lepidum* (n = 209, 67.42%) and *R. microplus* (n = 714, 78.55%). The nine tick species identified morphologically were also confirmed using molecular methods. However, during sequencing, two species (*Rhipicephalus appendiculatus and R. decoloratus*) had poor quality sequences and were excluded from the sequence analysis. Sequencing results indicated high nucleotide identity (96–100%) with sequences available in GenBank and Barcode of Life Database (BOLD). The phylogenetic analysis of partial mitochondrial COI and 16S rRNA gene sequences of ticks were used to confirm the morphological identification.

**Conclusion:**

The results showed a high burden of tick infestation on cattle, which could reduce animal production and potentially increase the risk of tick-borne diseases. Therefore, it is necessary to explore the epidemiological and molecular aspects of various tick species in other regions of Tanzania.

## Background

Ticks are one of the most important arthropod vectors and reservoirs for a wide variety of pathogenic agents such as viruses, bacteria, and protozoa which can cause diseases in human, livestock and wild animals [1]. Ticks transmit diseases that leads to extensive economic loses to resource-poor farming communities especially in tropical and subtropical regions where almost 80% of the world’s cattle population is reared [2, 3]. Tick-borne diseases (TBDs) such as East Coast fever, babesiosis, anaplasmosis and ehrlichiosis attribute to more than 70% of all cattle deaths in Tanzania as a result more than TSh. 72 billion is lost annually [4]. In addition to the transmission of infectious diseases, ticks are associated with great loss in milk production, meat production and hide quality [4].

Several ecological factors influence the tick prevalence and adaptation in different parts of the country. Abiotic factors such as soil moisture, humidity, soil pH, temperature and natural disasters and biotic factors e.g., host availability, vegetation caver, predators, parasites of ticks and the relations between individual tick species all affects the availability and diversity of ticks. The presence or absence of ticks is primarily dependent on humidity and moisture content of a local microclimate. Moreover, environmental conditions are continuously changing due to global warming, which may alter the distribution patterns and vectorial capacity of ticks [5].

Tanzania as an agricultural country, livestock related activities contribute 7.4% to the Gross Domestic Product (GDP) and the growth rate of the sector is low about 2.6% per annum [6]. About 65.7% of the households are involved in agricultural activities, 64.9% are engaged in crops only, while 33.3% engaged in crops and livestock and 2% in livestock only. The country has approximately 33.9 million cattle, 24.5 million goats and 8.5 million sheep, with about 90% of agricultural households keeping livestock of different kinds. Almost 95% of cattle populations in the country are reared under traditional agro-pastoral and pastoral husbandry systems. The National Livestock Policy recognizes that apart from contributing to the GDP, the livestock sector has a role to play in ensuring food security, as a source of income, providing farmers with employment and investment opportunities, providing draught power and manure for sustainable agriculture, and satisfying cultural roles [6]. The grazing land for the animals is no longer sufficient due to increased number of cattle, other domestic animals, and human populations. Most of the indigenous cattle are thus widely grazed in grasslands and woodlands and hence exposed to high risk of tick infestation [1, 7]. The climatic condition of Tanzania is greatly favoring the development and survival of several tick species.

Ixodid ticks of the genera *Rhipicephalus* (*R. appendiculatus, R. microplus, R. decoloratus*) and *Amblyomma* (*A. variegatum, A. lepidum and A. gemma*) are the most important and widely distributed tick species found in many parts of the country where cattle are raised [8, 9]. These tick species are important vectors of TBD pathogens reported in Tanzania and bordering countries. When compared to the studies in other countries, Tanzania’s data on tick epidemiology and genetic diversity is limited and insufficient [10]. Tick problems have been reported in Kilombero and Iringa Districts. Published studies related to tick infestation and species composition have been conducted in Ngorongoro [8], Iringa, Maswa [9], Mvomero [11], Rufiji [7], Mara, Singida, and Mbeya [1]. However, their information is limited to morphological characters. Studies have successfully demonstrated that mitochondrial DNA provides useful markers for studies on phylogenetic relationship of ticks. There is insufficient information on distribution and molecular characterization of ticks in southcentral Tanzania. Therefore, this study aimed to determine tick prevalence, distribution and infestation on cattle, at Kilombero and Iringa Districts of Tanzania.

## Materials and methods

### Description of the study area

**Fig. 1:**
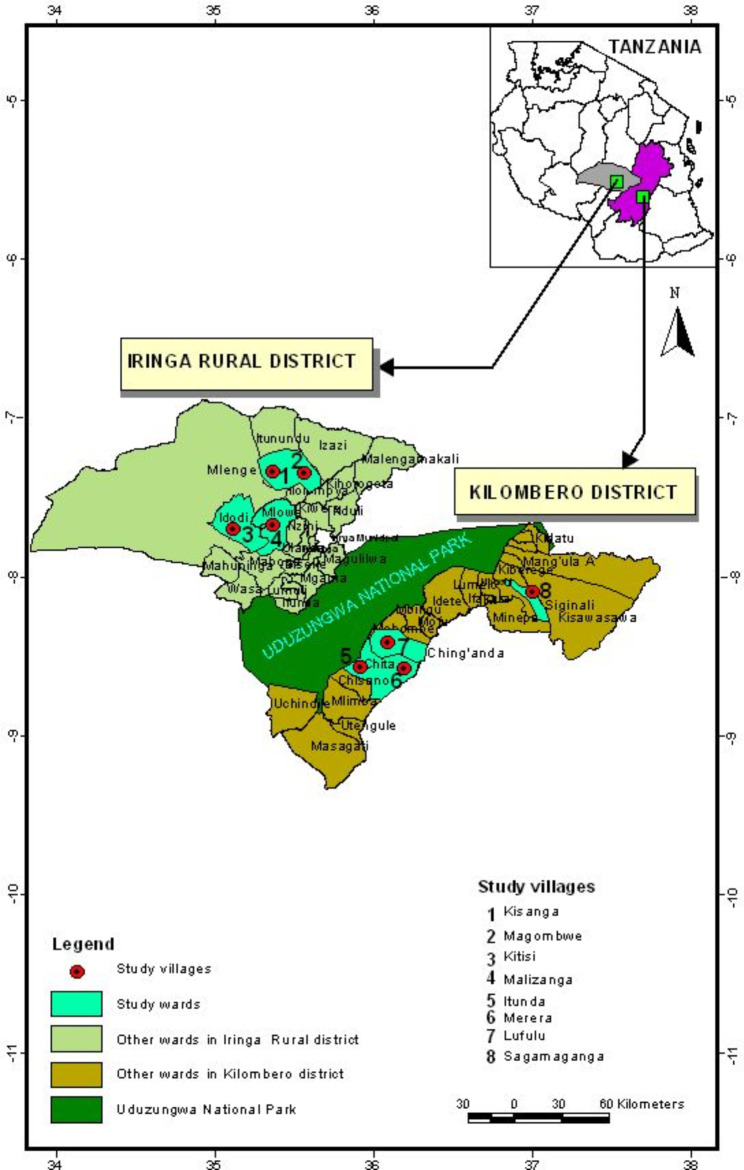
A map of Tanzania showing study areas. The red dots indicate the study communities both in Kilombero and Iringa Districts from which ticks were sampled

The study was conducted in Kilombero and Iringa Districts in Morogoro and Iringa regions in the Southern Highlands of Tanzania respectively. Kilombero District is one of the six administrative districts in Morogoro region and is located between 8 º 00’–16 º S and 36 º 04’–36 º 41’ E, with elevation ranging from 262 to 550 m above sea level and covering an area of 14,246 km^2^ in the region [12]. The climate is marked by a rainy season from November to May and a dry season from June to October, with annual rainfall ranging from 1200 to 1800 mm. Average annual temperatures in Kilombero District range from 26 to 38 °C. The cattle population was about 157,000 cattle [6]. The sampling villages in Kilombero District were Merera, Idunda, Sagamaganga and Lufulu (Fig. 1). Iringa District, one of the seven administrative districts in Iringa region is located between 7 º 46’23.14’’ S and 35 º 41’ 56.83’’ E and covering an area of 20,576 km^2^, with an elevation ranging from 900 to 2300 m above sea level. The climate is marked by a rainy and cooler season from November to May and a dry and cool season from June to September. It generally receives annual rainfall of about 500–1600 mm. The average annual temperature in Iringa District ranges from 20 to 25 ºC. The lowland zone in Iringa District is characterized by low mean annual rainfall of about 500–600 mm, and temperatures of about 20–25 °C [6]. The land is mostly occupied by the National parks, forests, rocky mountains and water bodies. The district had the third largest number of cattle in the region and they were nearly all indigenous. The population of cattle was about 150,810 cattle. Most of the animals were indigenous reared under traditional system, largely free grazing and tethering. The sampling villages in Iringa District were Magombwe, Kisanga, Kitisi and Malizanga (Fig. 1).

### Sample size estimation

Sample size was estimated by using CDC Epi info software version 7.2.4.0 adapting the following formula:

n = ((Z_α /2_)^2^ x pq) ÷ d^2^, where; n = required sample size; Z _0.033 / 2_ = Z _0.0165_ = 1.96 at 96.7% confidence interval; α = probability of type 1 error (0.05 sided); p = estimated prevalence of cattle with ticks (30%) [1]; q = Power (1 – p); d = Margin of error for expected confidence level (3.3%). Based on this software, the minimum required sample size was 739 cattle of all ages and sexes present in the study areas.

### Study design and sampling method

A repeated cross-sectional study design using qualitative and quantitative methods of data collection and analysis was adopted. Tick sampling was performed both in wet (January and May) and dry (July and August) seasons of the year 2021. The two districts were purposively selected as they are bordering the wildlife conservation areas and existing previous engagements with the villages during the past health for animal and livelihood improvement studies. The eight study villages (four from each district) were also purposively selected. Three herds having at least 40 cattle were randomly selected from each village with the assistance of Livestock Extension Officers (LEOs) providing the information on areas where pastoralists have settled with their animals.

### Tick collection and counting

During field visits twenty cattle were randomly selected from each herd and manually restrained by the help of herdsmen to allow physical examination and tick inspection [13]. During tick inspection, the animal’s body was marked into five body zones; zone 1 (Head, ears, flap of skin on lower surface of neck and ventral surface of thorax), zone 2 (Back surface of the body), zone 3 (Side of main body and area between forelegs and body), zone 4 (Groin, flank, abdomen and around inner thigh of the hind legs), zone 5 (Perineum including areas between anus and genital organs). The animals were then visually inspected for ticks all over the body sites. Additional information on animal health status (body condition score), age, and sex was recorded, along with frequency of tick control and tick control methods used in each household were recorded [1]. Tick burden on each animal was obtained by counting the number of ticks from one side of the body and the results were multiplied by two to represent the tick burden on the whole body of the animal as described by Rehman [3]. Ticks were then removed from different predilection sites of the animal’s body marked as zone 1–5 using blunt ended forceps [14]. The forceps were used to grip the tick firmly over its scutum and mouthparts as closely to the host skin as possible, then pulled strongly and directly out of the skin. All ticks from each zone were pooled together and transferred into respective empty 15 ml falcon tube and kept in a dry shipper to immobilize the ticks until sorting at the Health for Animal and Livelihood Improvement (HALI) project molecular diagnostic laboratory at the College of Veterinary Medicine and Biomedical Sciences, Sokoine University of Agriculture.

### Morphological identification of ticks

The preserved ticks were taken from − 80 °C freezer, rinsed with 70% ethanol, then distilled water, followed by brief drying on paper towel. The ticks were sorted by sex and genera based on the presence or absence of banded legs, colored or patterned scutum and conscutum, presence or absence of festoons and eyes and shapes of the mouthparts using magnifying hand lens. The sorted ticks were then identified using published morphological keys for African ticks [15, 16]. The ticks were further morphologically examined to species on a stereomicroscope (Brunel Stereomicroscope Ltd, UK) by experienced laboratory personnel at the Parasitology laboratory in the Department of Veterinary Microbiology, Parasitology and Biotechnology at Sokoine University of Agriculture.

### DNA extraction and molecular identification of ticks

The DNA was extracted from 42 ticks, (1–5 ticks from each species) randomly selected from the nine tick species initially identified by morphological characters. The DNA extraction was performed using Quick-DNA Minprep Plus Kit (D4068, Zymo Research, CA, USA) according to manufacturer’s instructions. The extracted DNA was eluted in 50 µl of DNase/RNase-free water and stored at − 80ºC for subsequent use for PCR [17–19]. The tick species were confirmed using conventional PCR targeting Mitochondrial Cytochrome c Oxidase subunit 1 (CO1) or 16S rRNA as DNA barcoding genes for selected members of tick species and those which were found difficult to identify to species level morphologically [13, 20, 21].

The fragment of CO1 gene was amplified using primer sets Cox1-F- (5’- GGA ACA ATA TAT TTA ATT TTT GG-3’) and Cox1-R- (5’-ATC TAT CCC TAC TGT AAA TAT ATG − 3’) amplifying approximately 820 bp [22]. The 16S rRNA gene was amplified using primer sets T16S-F- (5’-TTA AAT TGC TGT RGT ATT-3’) and T16S-R- (5’-CCG GTC TGA ACT CAS AWC-3’) amplifying approximately 455 bp [23]. The PCR amplifications were performed in a total reaction volume of 25 µl containing 2.5 µl of 10X PCR buffer, 0.75 µl of 50 mM MgCl_2_, 0.5 µl of 10 mM dNTPs, 1.0 µl (10 µM) of each forward and reverse primer, 0.10 µl of Platinum Taq DNA Polymerase (Invitrogen, Carlsbad, CA, USA), 17.15 µl of Molecular grade water and 2 µl of template DNA in a thermal cycler (SimpliAmp thermocycler, Applied Biosystems, Thermo Fisher Scientific Inc). The cycling conditions for CO1 were as follows: 95 °C for 5 min, followed by 45 cycles of 95 °C for 30 s, 54 °C for 1 min, 72 °C for 1 min and then 72 °C for 5 min [21]. For the 16S rRNA, the cycling conditions were as follows: 94 °C for 5 min, followed by 45 cycles of 94 °C for 30 s, 50 °C for 45 s, 72 °C for 45 s and then 72 °C for 7 min [23]. A negative control with ddH_2_O in place of DNA was included in each run. The obtained PCR products were separated on a 1.5% (w/v) agarose gel in 1x TBE buffer (SERVA Electrophoresis, Heidelberg, German) stained with gel red (Phonex Research Products, Candler, USA) and viewed under UV transilluminator.

The PCR products of CO1 and 16S rRNA fragments were then sequenced using forward and reverse primers used to generate the PCR products. The sequencing reactions were performed in the DNA Master cycler pro-384 (Eppendorf) using BigDye Terminator Cycle Sequencing Kit version 3.1 (Applied Biosystems, Foster City, CA) following the protocols supplied by the manufacturer. The fluorescent-labeled fragments were purified using the BigDye XTerminator Purification Kit (Applied Biosystems, Foster City, CA). The samples were run for electrophoresis in an ABI 3730xl DNA Analyzer (Applied Biosystems, Foster City, CA).

### Data management and statistical analysis

All data collected in this study were stored in a computer using Microsoft excel software version 2109 where they were sorted and checked for completion before doing statistical analysis. The age of cattle was grouped into calves (≤ 6 months), juveniles (7 to 24 months) and adults (> 24 months) [1, 24]. Cattle health status was evaluated using Body Condition Score (BCS). The BCS was categorized into poor (BCS 1 and 2), average (BCS 3) and good (BCS of 4 and 5) [1, 24]. The study prevalence of tick infestation was determined by dividing the number of infested cattle by total number of cattle examined and was expressed as percentage. Descriptive statistics on tick prevalence data was performed using Epi Info software version 7.2.4 (CDC, Atlanta, USA) to compare the difference in tick species proportions between the study areas and seasons. Fisher’s exact test was performed to detect the difference between the proportion of hard tick species and the study areas and seasons. Additionally, the tick species count was used as dependent variable while district and season were used as independent variables for performing a one-way ANOVA to compare mean tick burden between variables (e.g., Cattle age groups, BCS and frequency of tick control). The *p*-value (0.05) was considered statistically significant in all statistical tests.

The obtained DNA sequences were compared with sequences on GenBank database using Basic Local Alignment Search Tool (BLAST) to obtain sequence similarities (https://blast.ncbi.nlm.nih.gov (accessed on 23 December 2021)). The quality of sequencing chromatogram was checked using Sequence Scanner Version 2.0 software (Applied Biosystems, Foster City, CA). The reverse complement and forward nucleotide sequences delimited by reverse and forward primers sequence were aligned to obtain a consensus nucleotide sequence using Bioedit version 7.2.5 (Ibis Biosciences, Carlsbad, CA). The consensus nucleotide sequence was used in BLASTn to search for nucleotide identity in comparison with available nucleotide sequences at GenBank database. The nucleotide sequences were then analysed using Molecular Evolutionary Genetics Analysis (MEGA) X software [25] and aligned using Clustal-W to determine the similarity between the sequences. In addition, representative CO1 and 16S rRNA tick sequences from previous studies were downloaded from GenBank for phylogenetic analysis. Multiple sequence alignments were performed and the neighbor-joining (NJ) method was used to construct the cladogram tree in MEGA X [25–28]. The evolutionary distances were computed using the Maximum Composite Likelihood method and were in the units of the number of base substitutions per site. All ambiguous positions were removed for each sequence pair. The confidence values for individual branches of the resulting trees were determined through bootstrap values with 1000 replicates to statistically support the nodes on the tree.

### Ethical approval

The ethical approval for this research was obtained from Ethical Committee of the Sokoine University of Agriculture, permit number SUA/ADM/R.1/8A/734_15/02/2021. The permission to carry out this study in the respective study sites was granted by the local government through District Veterinary Officers in Kilombero and Iringa Districts. Animal owner’s consents were sought verbally prior to data collection. The study was conducted with full approval from household owners, district councils of the study areas and the Sokoine University of Agriculture.

## Results

A total of 740 cattle were examined for tick infestation, 45.68% (n = 338) from Kilombero District and 54.32% (n = 402) from Iringa District. An overall tick prevalence of (41.08%, n = 304) was recorded in the study areas. The tick prevalence in Kilombero District was (41.42%, n = 140) and (40.80%, n = 164) in Iringa District, both were nearly equal to the overall tick prevalence and no significant difference observed (*p* > 0.05) (Table 1). In total 1,889 ticks were collected from the infected cattle whereas, after counting ticks on one side of the animal’s body and doubled a total of 3,560 ticks were recorded. Seasonally, tick infestation prevalence was (41.11%, n = 148) during the wet season and (41.05%, n = 156) during the dry season (Table 1). Tick infestation prevalence was higher in male (44.02%, n = 92) than female cattle (39.92%, n = 212). Based on cattle age groups, tick infestation prevalence was highest in calf (51.61%, n = 16), followed by adult (40.91%, n = 216) and juvenile (39.78%, n = 72). Using Body Condition Score, tick infestation was highest on cattle with average health condition (41.78%, n = 216), and least in poor (39.82%, n = 45) and cattle with good health condition (39.09%, n = 43). Moreover, tick infestation prevalence was almost equal between the tick control frequency categories (Table 1). Therefore, there was no statistically significant difference in tick infestation prevalence between the two seasons, cattle sex, age groups, health categories and tick control frequencies observed in this study *p* > 0.05.

Of all tick species identified, *Rhipicephalus microplus* had the highest prevalence (48.1%, n = 909), followed by *Rhipicephalus evertsi* (16.4%, n = 310) and *Amblyomma lepidum* (16.4%, n = 310) while, *Hyalomma albiparmatum* had the lowest prevalence (0.2%, n = 3) (Table 2). Season wise, *Rhipicephalus microplus* was recorded with the highest proportions during the wet season 59.8% and 35.2% during dry season, followed by *A. lepidum* which had a higher proportion 19.3% during dry and 13.8% during wet season, and *R. evertsi* had a higher proportion 20.8% during dry compared to 12.4% during wet season. The least tick proportion was observed in *H. albiparmatum* with a proportion of 0.3% during wet and 0% during dry season (Table 3). In general, there was statistically significant difference between the seasons on the proportion of *R. microplus*, *R. evertsi*, *R. appendiculatus*, *H. rufipes*, and *A. lepidum* (*p* < 0.05) (Table 3). On the other hand, *Amblyomma gemma*, *A. variegatum* and *R. decoloratus* had a higher proportion during wet than dry season however, the difference was not significant (*p* > 0.05) (Table 3).

For the case of mean tick burden, a higher overall mean tick burden of 12.07 ± 0.91 was observed in Kilombero compared to 11.40 ± 1.00 in Iringa District, however, the difference was not statistically significant (CI = 95%, *p* = 0.640). Mean tick burden on cattle was higher during wet than dry season however the difference was not statistically significant (*p* = 0.436) (Table 4).

**Table 1:** Number of cattle examined, infested cattle and tick infestation prevalence with respect to district, season, cattle sex, age, animal health status and tick control frequency

Variables	Number of cattle examined	Number of cattle infested	Prevalence (%)	*p*- value
District				
Iringa	402	164	40.80	0.864
Kilombero	338	140	41.42	
Season				
Dry	380	156	41.05	0.987
Wet	360	148	41.11	
Cattle sex				
Female	531	212	39.92	0.308
Male	209	92	44.02	
Cattle age group				
Adult	528	216	40.91	0.240
Calf	31	16	51.61	0.217
Juvenile	181	72	39.78	0.789
Cattle health status				
Average	517	216	41.78	0.603
Good	110	43	39.09	0.911
Poor	113	45	39.82	0.702
Tick control frequency				
Weekly	135	56	41.48	0.844
Biweekly	294	119	40.48	0.909
Monthly	154	65	42.21	0.901
Occasionally	133	54	40.60	0.884
Unknown	24	10	41.67	0.987

**Table 2:** Sex ratio and prevalence for the identified tick species collected on cattle from Kilombero and Iringa Districts. Male (M); Female (F)

Tick species	Males	Females	M: F	Total ticks	Prevalence (%)
*Amblyomma gemma*	88	32	2.6:1	120	6.4
*Amblyomma lepidum*	247	63	3.9:1	310	16.4
*Amblyomma variegatum*	14	15	1:1.1	29	1.5
*Hyalomma albiparmatum*	3	0	3:0	3	0.2
*Hyalomma rufipes*	32	18	1.8:1	50	2.6
*Rhipicephalus appendiculatus*	45	95	1:2.1	140	7.4
*Rhipicephalus decoloratus*	0	18	0:18	18	1.0
*Rhipicephalus evertsi*	188	122	1.5:1	310	16.4
*Rhipicephalus microplus*	63	846	1:13.4	909	48.1

**Table 3:** The proportion and counts of hard ticks from Kilombero and Iringa Districts during dry and wet season

	Season				District		
	Dry (%)	Wet (%)	*p*-value		Iringa (%)	Kilombero (%)	*p*-value
**Tick genera**							
*Amblyomma*	235 (51.2)	224 (48.8)	0.021		447 (97.4)	12 (2.6)	0.000
*Hyalomma*	32 (60.4)	21 (39.6)			53 (100.0)	0 (0.0)	
*Rhipicephalus*	630 (45.8)	747 (54.3)			501 (36.4)	876 (63.6)	
**Tick species**							
*A. gemma*	50 (5.6)	70 (7.1)	0.219		119 (99.2)	1 (0.8)	0.000
* A. lepidum*	173 (19.3)	137 (13.8)	0.001		306 (98.7)	4 (1.3)	0.000
* A. variegatum*	12 (1.3)	17 (1.7)	0.576		22 (75.9)	7 (24.1)	0.014
* H. albiparmatum*	0 (0.0)	3 (0.3)	0.252		3 (100.0)	0 (0.0)	0.252
* H. rufipes*	32 (3.6)	18 (1.8)	0.021		50 (100.0)	0 (0.0)	0.000
*R. appendiculatus*	121 (13.5)	19 (1.9)	0.000		137 (97.9)	3 (2.1)	0.000
*R. decoloratus*	6 (0.7)	12 (1.2)	0.246		14 (77.8)	4 (22.2)	0.055
*R. evertsi*	187 (20.8)	123 (12.4)	0.000		306 (98.7)	4 (1.3)	0.000
*R. microplus*	316 (35.2)	593 (59.8)	0.000		44 (4.8)	865 (95.2)	0.000

**Table 4:** Number of cattle infested, total number of ticks, mean tick burden per cattle ± standard error of mean (SE) with respect to district, season, cattle sex, age, animal health status and tick control frequency

Variables	No. of cattle	Tick counts	Mean tick burden ± SE	Std Dev	*p-*value
District					
Iringa	164	1870	11.40 ± 1.00	12.76	0.625
Kilombero	140	1690	12.07 ± 0.91	10.80	
Season					
Dry	156	1746	11.19 ± 0.99	12.33	0.436
Wet	148	1814	12.26 ± 0.92	11.40	
Cattle sex					
Female	212	2286	10.78 ± 0.73	10.66	0.039
Male	92	1274	13.85 ± 1.47	14.14	
Cattle age group					
Adult	216	2654	12.29 ± 0.85	12.44	0.403
Calf	16	160	10.00 ± 2.98	11.91	
Juvenile	72	746	10.36 ± 1.18	10.02	
Cattle health status					
Average	216	2394	11.08 ± 0.75	11.01	0.221
Good	43	624	14.51 ± 2.21	14.44	
Poor	45	542	12.04 ± 1.95	13.09	
Tick control frequency					
Weekly	56	338	6.11 ± 0.77	5.75	0.000
Biweekly	119	1 516	12.77 ± 1.01	11.02	
Monthly	65	970	14.83 ± 2.20	17.74	
Occasionally	54	550	10.15 ± 0.95	6.99	
Unknown	10	186	18.60 ± 2.91	9.19	

Among the ticks collected from Kilombero District, *Rhipicephalus microplus* was the most abundant tick species (97.4%, n = 865) while in Iringa District, *A. lepidum* and *R. evertsi* were the most abundant species of all ticks collected each (30.6%, n = 306). In addition, *Hyalomma albiparmatum* (100%, n = 3) and *H. rufipes* (100%, n = 50) were only recorded in Iringa District while none was recorded in Kilombero. For *H. rufipes*, the difference in proportion between the two districts was statistically significant (*p* < 0.05). The other six tick species including, *A. gemma, A. lepidum, A. variegatum, R. appendiculatus, R. decoloratus* and *R. evertsi* were all recorded with significantly higher proportions in Iringa District compared to Kilombero District (*p* < 0.05) (Table 3). *Rhipicephalus microplus* was the only species recorded with significantly higher proportion in Kilombero District than in Iringa District.

With regards to cattle sex, age group, health status and tick control frequency, a significantly higher mean tick burden of 13.85 ± 1.47 was recorded in male as compared to female cattle 10.78 ± 0.73, (*p* < 0.05) (Table 4). The highest mean tick burden 12.29 ± 0.85 was recorded in adult cattle (> 24 months) as compared to calves ( < = 6 months) and juvenile (7–24 months) which had mean tick burden of 10.00 ± 2.98 and 10.36 ± 1.18 respectively. In general, there was no statistically significant difference in mean tick burden between the cattle age groups (*p* = 0.403) and between the cattle health status groups (*p* = 0.221). Based on tick control frequency categories, a significant low mean tick burden (6.11 ± 0.77) was recorded on cattle reported with weekly tick control frequency (*p* < 0.001) (Table 4).

With regard to tick distribution, there was higher tick species diversity in Iringa than Kilombero District (Table 5). However, in Kilombero District, *R. microplus* was highly distributed in all sampled villages as compared to Iringa District. Among the five predilection sites on cattle’s body, ticks were distributed in all the five body zones. Tick distribution was highest on zone 4 (56.11%, n = 1,060) which includes (groin, flank, abdomen and around inner thigh of the hind legs), followed by zone 5 (23%, n = 451) and least on zone 2 (0.74%, n = 14) which includes back surface of the body. *Amblyomma lepidum* and *Rhipicephalus microplus* species were distributed in all the five body zones and recorded with the highest proportions on zone 4 (including, *A. lepidum* (67.42%, n = 209) and *R. microplus* (78.55%, n = 714) (Table 6).

**Table 5:** The distribution of the tick species collected on cattle from villages in Kilombero and Iringa Districts

	Iringa					Kilombero			
Tick species	Kisanga	Kitisi	Magombwe	Malizanga		Lufulu	Idunda	Merera	Sagama*
*A. gemma*	45	23	5	46		0	0	1	0
*A. lepidum*	88	6	202	10		0	0	4	0
*A. variegatum*	2	9	9	2		0	0	2	5
*H. albiparmatum*	0	0	1	2		0	0	0	0
*H. rufipes*	27	7	14	2		0	0	0	0
*R. appendiculatus*	4	25	0	108		0	0	0	3
*R. decoloratus*	6	0	8	0		1	1	0	2
*R. evertsi*	104	32	154	16		0	0	4	0
*R. microplus*	2	3	39	0		119	299	293	154

*Name shortened: Sagama* (Sagamaganga)

**Table 6:** The distribution of hard tick species identified with respect to cattle body zones

	Cattle body zones (Tick predilection site on cattle)				
Tick species	Zone 1	Zone 2	Zone 3	Zone 4	Zone 5
* A. gemma*	6	0	36	59	19
*A. lepidum*	11	3	60	209	27
*A. variegatum*	0	4	12	10	3
*H. albiparmatum*	1	0	0	2	0
*H. rufipes*	0	0	2	3	45
*R. appendiculatus*	103	0	4	27	6
*R. decoloratus*	0	2	1	6	9
*R. evertsi*	4	0	2	30	274
*R. microplus*	12	5	110	714	68
Total ticks n (%)	137 (7.25)	14 (0.74)	227 (12.02)	1,060 (56.11)	451 (23.88)

For molecular identification of tick species, a total of 42 representative ticks, (1–5 ticks) from each species were randomly selected for molecular analysis. The nine tick species identified morphologically were also identified by molecular methods however, during sequencing two species (*Rhipicephalus appendiculatus and R. decoloratus*) had poor quality sequences and were excluded from the analysis. The CO1 gene was successfully amplified from 92.86% (n = 39) of the selected-on host ticks. The 16S rRNA gene was successfully amplified from 100% (n = 8 including 3 of the samples with unreliable CO1 results and 5 more samples that were successfully amplified by CO1). The amplification of approximately 455 bp sequence of 16S rRNA produced the expected amplification products. The nucleotide sequences of Tanzanian ticks obtained from this study were submitted at the GenBank and provided with accession numbers (OM974109 - OM974112 and OM978262 - OM978265).

Based on the CO1 gene sequences, *Amblyomma gemma* from this study (GenBank accession no. OM974111) was 100% homologous to an *A. gemma* isolate sequence from Kenya (BOLD: ARAK131-13). The *A. lepidum* sequence (GenBank: OM974112) from this study was most similar to an *A. lepidum* isolate sequence from Kenya (GenBank: KP987775, 99.57% homology). *Hyalomma albiparmatum* sequence from this study (GenBank: OM974110) was most closely related to a *H. albiparmatum* sequence from Israel (GenBank: KU130576, 96.08% homology), whereas, the *H. rufipes* sequence from this study (GenBank: OM974109) was most closely related to a *H. rufipes* sequence from France (GenBank: KX000643, 99.74% homology).

Based on the 16S rRNA gene sequences, *Rhipicephalus evertsi* sequence from this study (GenBank: OM978262) had the closest relationship (100%) to a *R. evertsi* sequence from Zambia and Tanzania (GenBank: LC634571 and MN961124). *Amblyomma variegatum* from this study (GenBank: OM978264) had the closest similarity (99.47%) to an *A. variegatum* sequence from Ethiopia (MN150175) respectively. Lastly, the 16S rRNA gene sequence of *R. microplus* from this study (GenBank: OM978265) was most closely related (100% homology) to a *R. microplus* sequence from Uganda and Colombia (GenBank: KY688461 and MN650726) respectively.

**Fig. 2:**
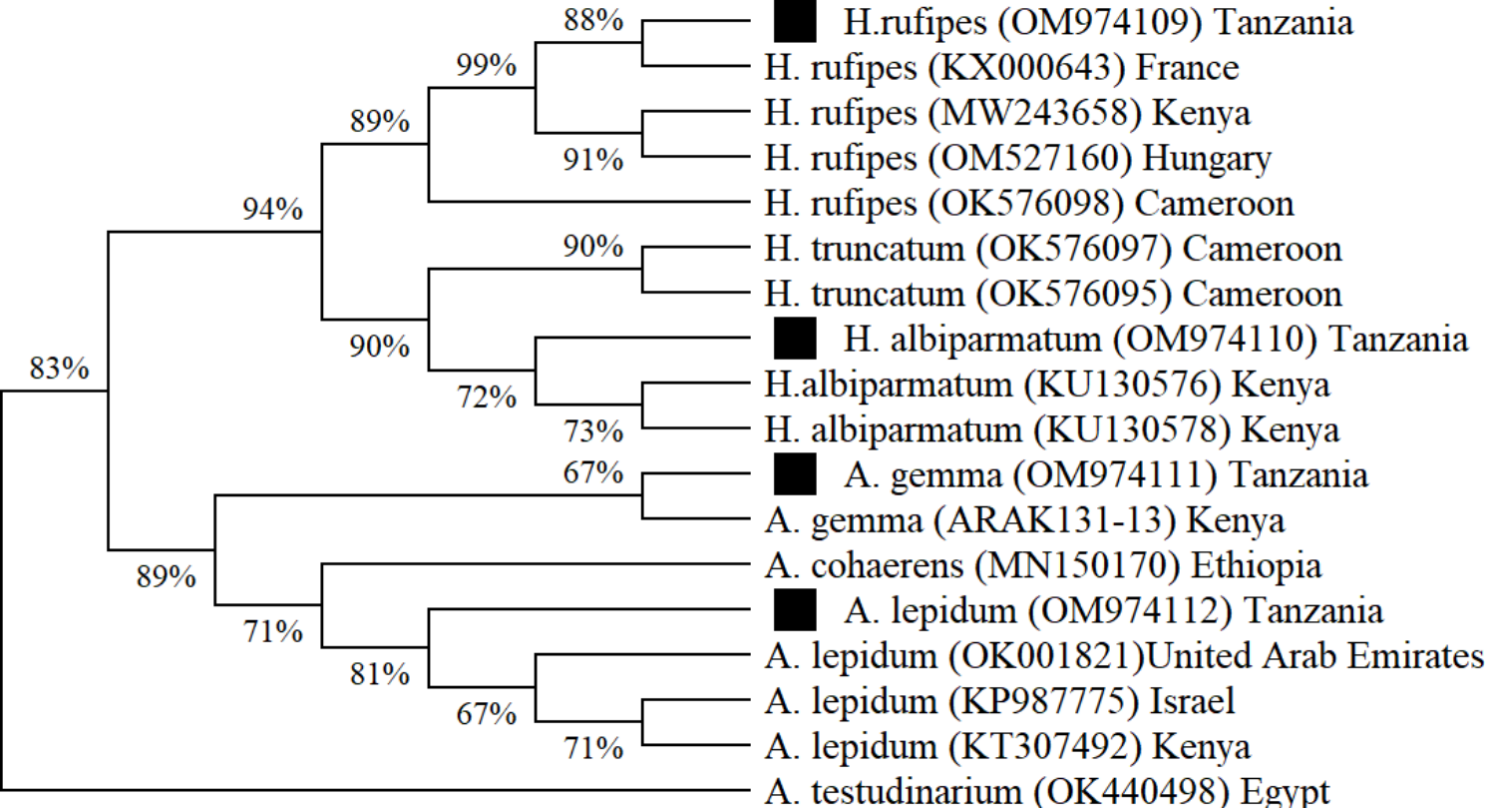
Neighbor-Joining cladogram tree based on ticks mitochondrial CO1 gene sequences. The percentage of replicate trees in which the associated taxa clustered together in the bootstrap test are shown next to the branches. Black squares represent samples sequenced in this study

**Fig. 3:**
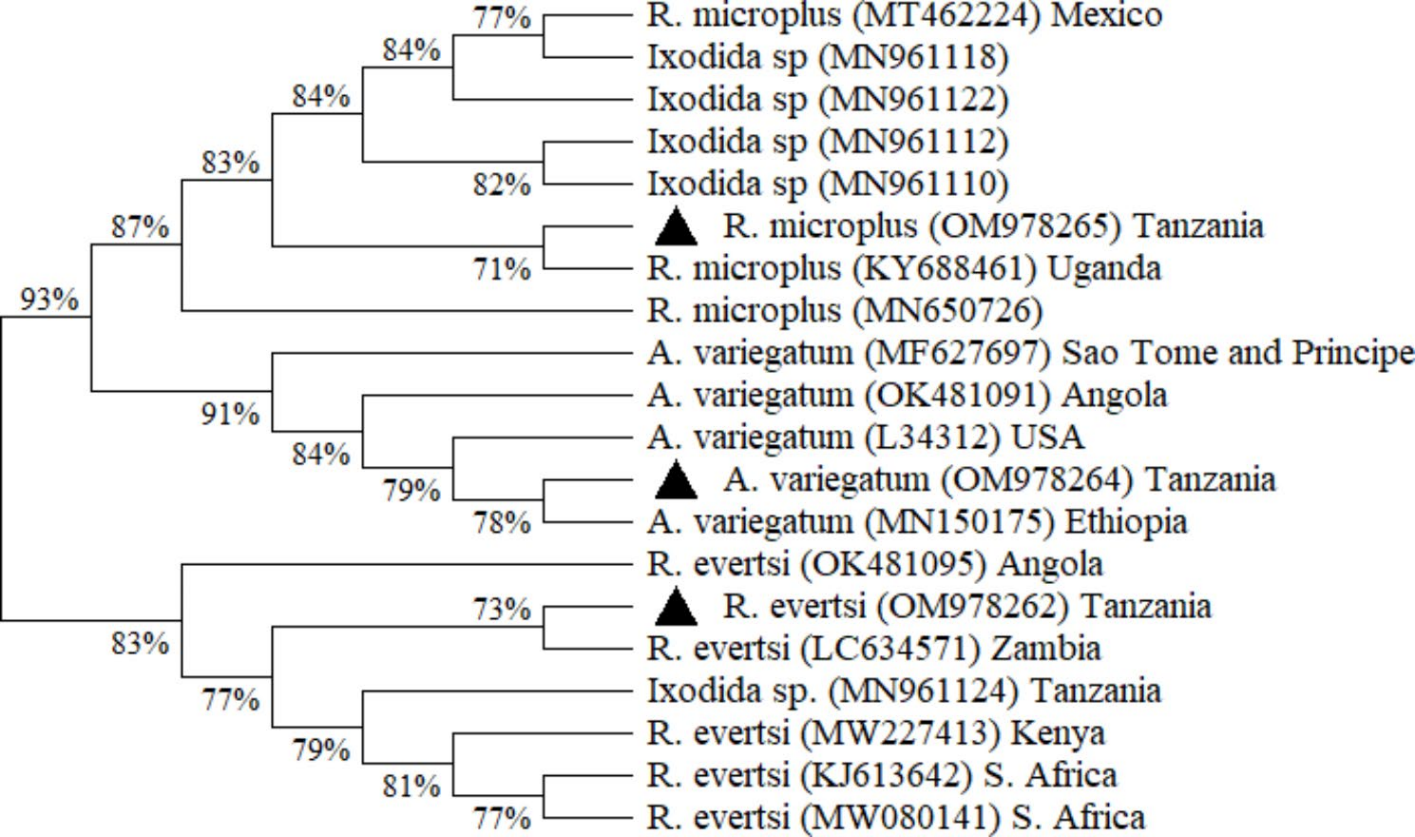
Neighbor-Joining cladogram tree based on ticks 16S rRNA gene sequences. The percentage of replicate trees in which the associated taxa clustered together in the bootstrap test are shown next to the branches. Black triangles represent samples sequenced in this study

Phylogenetic analysis based on mitochondrial CO1 and 16S rRNA nucleotide sequences of the identified tick species was performed to determine the genetic relationship between the nucleotide sequences obtained in this study and reference sequences obtained from GenBank. Alignment of CO1 gene nucleotide sequences obtained from each tick species in this study showed that the sequences were 100% identical. Similarly, the 16S gene nucleotide sequences obtained from each tick species were found to be 100% identical. Therefore, a single sequence from each tick species was selected for phylogenetic analysis. In both mitochondrial CO1 (Figs. 2) and 16S rRNA (Fig. 3) cladogram trees, four major clusters were observed with all the nodes strongly supported by high bootstrap values.

## Discussion

In this study ticks were prevalent in the study areas and a high overall tick prevalence was reported. The presence of tick species and the high tick infestation prevalence in the study has been reported in other areas of Tanzania and may be attributed to unrestricted cattle movement from one area to another for water and pasture and cattle trade, which is a common phenomenon across the country. Similar findings on tick prevalence were reported in previous studies [29]. The similar tick prevalence between the two districts could be due to similar agroecological setting and animal husbandry practices including strategies and awareness of the farmers on application of acaricides through hand spraying approaches [24, 30]. In this study, season had no significance effect on tick prevalence, suggesting that cattle are susceptible to tick infestation during both wet and dry season. In the current study cattle sex had no significance effect on tick prevalence on cattle, however the slightly higher prevalence on males may signify that male cattle are less resistant to ticks than females, and this could possibly be attributed to influences of testosterone in males, which reduce innate and acquired resistance to tick feeding [31].

The findings from this study suggest that male cattle could be more likely challenged by tick infestation as a result of high tick prevalence. Age group had no significance effect on tick prevalence on cattle. Although cattle age group was not significantly associated with tick prevalence, it was observed that calves were slightly more susceptible to tick infestation than other age groups. In the current study animal health group had no significance effect on tick prevalence on cattle, suggesting an equal tick challenge by tick infestation to all cattle health groups. This could be due to the fact that all the animals from the health categories walk for long distance to be grazed in the field and kept together at home, as a result all groups were equally susceptible to tick infestation. Similar findings among cattle health groups was reported in previous studies [1].

In this study, tick control frequency had no significance effect on tick prevalence on cattle. Although the use of acaricides was not significantly associated with tick prevalence in this study, cattle from herds where acaricides were used on bi-weekly basis had the least tick infestation prevalence. Ticks were found in all cattle herds suggesting that acaricide resistance could occur. Findings from this study suggest an equal challenge by tick infestation to all tick control frequency groups.

A significantly higher proportion of *R. microplus* was recorded in Kilombero District (95.16%, n = 865) as compared to Iringa District. This difference may be due to favorable climatic condition in Kilombero, which is marked by annual rainfall ranging from 1,200 to 1,800 mm and average annual temperatures ranging from 26 to 38 °C. In addition, the study area lies along Kilombero Valley in the lowlands ranging in elevation from 270 to 300 m asl. The households are also scattered and dispersed in the study areas, which may limit the interaction between the animal herds and result in a lower distribution of other ixodid ticks. Similar findings has been reported in various part of Tanzania [7, 32]. Previous studies in Sudan have shown that this tick occurs in humid localities with steppe areas that have hot, dry seasons [15]. *Rhipicephalus microplus* and *R. decoloratus* are important vectors of *Babesia bigemina* and *Babesia ovis* that causes bovine babesiosis. Moreover, they are vectors of *Anaplasma marginale,* that causes bovine anaplasmosis. However, *R. microplus,* in terms of control management is known to be more resistant to many acaricides. These two species rarely occur together due to interspecies competition, despite their similarity in temperature and rainfall requirements [1].

The tick species in this study were present both in dry and wet seasons, except *H. albiparmatum* which was only found during the wet season. *Amblyomma lepidum, H. rufipes, R. appendiculatus* and *R. evertsi* were both prevalent on cattle during the dry season while, *A. gemma*, *A. variegatum*, *R. decoloratus* and *R. microplus* were more prevalent on cattle during the wet season. The results show that these ticks can maintain themselves under certain condition and perhaps occur seasonally. Findings from this study have showed a clear seasonal variation demonstrated by *R. microplus*, with significantly high infestation occurring during the wet season [7]. This suggests that environmental factors such as rainfall, temperature and relative humidity have great influence on the population of ticks as observed in the previous reports from Tanzania [33, 34]. The absence of significant difference in proportions of the other three species including, *A. gemma*, *A. variegatum* and *R. decoloratus* between the seasons suggests that their activities are less affected by weather parameters [35]. The few *H. albiparmatum* collected indicate that climatic conditions and other unknown factors in Kilombero and Iringa Districts are not favorable for this tick species to be able to establish itself and adopt the climatic conditions.

The small difference in tick abundance observed between the two districts could be due to similar agroecological setting and animal husbandry practices including strategies and awareness of the farmers on application of acaricides through hand spraying techniques [24, 30]. Likewise, such mean tick burden of Ixodid ticks were reported from different parts of the country in Mara region (35.80 ± 4.30), Singida (12.9 ± 2.10) and Mbeya (7.0 ± 0.40) [1].

The higher mean tick burden per animal during the wet season could be due to high humidity and low temperature range that facilitate the growth and survival of ticks at their different stages of life. Similar findings have been reported in the previous studies [36, 37]. The higher tick infestation in male cattle may signify that male cattle are less resistant to ticks than females, and this could possibly be attributed to testosterone in males, which reduce innate and acquired resistance to tick feeding [31]. The findings from this study suggest that female cattle are less likely challenged by tick infestation as a result of low mean tick burden. Similar findings have been reported in the previous studies [38]. These findings in contrast are not consistent with studies reported in Mara, Singida and Mbeya regions, Tanzania [1], Pakistan [3] and Eastern Ethiopia [39].

Age group had no significant effect on mean tick burden per animal (*p* > 0.05). Finding from this study suggest an equal challenge by tick infestation to both calves, juvenile and adult cattle. Adult and juvenile cattle were grazed in grasslands and bushy areas located typically far away from the households. The calves usually do not graze with adult cattle, but rather graze near the household dwellings, thus perhaps reducing their chance of contacting ticks. Similar findings have also been reported in Ngorongoro, Tanzania [37], Central Nigeria [1, 36] where calves were grazed separately from adults.

In the present study, no significant effect of cattle health status was observed on mean tick burden per animal (*p* > 0.05). This could be due to the fact that all the animals walk long distance to be grazed in the field and kept together at home, as a result all groups are equally susceptible to tick infestation. Similar findings among cattle health groups was reported in Mbeya region, Tanzania [1]. This was not consistent with previous studies in Ethiopia [40]. The significantly lower mean tick burden observed on the cattle reported with weekly tick control frequency could be attributed to the frequent application of acaricide to control ticks on cattle by hand spraying. A similar observation was reported in Mara and Mbeya regions, Tanzania [1]. However, when using hand spraying method, the acaricides do not reach the hidden parts of the animal body, as a result not all ticks are killed [41]. Acaricide application and other means of tick control like rotational grazing, hand picking, pasture burning and many others are economically viable methods for controlling ticks and TBDs on cattle and reduces tick burden, however their practicability on a large scale is limitted [11].

The tick species found in this study were highly diverse and widely distributed in the Iringa District as compared to the Kilombero District. This could be attributed to the average annual temperature in Iringa which ranges from 20–25ºC, a low mean annual rainfall of about 500–600 mm and the elevation that ranges from 718 to 945 m asl in the sampled areas which favors the reproduction of these tick species [8]. Furthermore, the area was characterized with trees, short shrubs and grass cover which could be favorable for the survival of the ixodid ticks [8]. In addition, extensive livestock grazing practices put more pressure on the land resources which results in the need of continuous movement of large number of livestock in search of water and pasture. This often brings livestock to share the pasture with wild animals in the wildlife-livestock interface ecosystem bordering the conserved area of Ruaha National Park [42–44]. Previous studies have also reported the presence of ixodid ticks in cattle and wild animals in Iringa Municipality [9]. Ixodid ticks have been reported in livestock and wild animals such as zebra, buffalo, elephant, leopard, antelope and warthog [9, 45]. These findings suggest that these ticks could be predominantly found on animals that live in and around wildlife-livestock interface bordering the Ruaha National Park. As a result, the above factors could have promoted the great diversity and distribution of the Ixodid tick species in the study area. The lower numbers of *H. albiparmatum* has been previously reported in some parts of Tanzania [8, 34].

The highest number of ticks were located on zone 4 of the animals which includes the groin, flank, abdomen, and around inner thigh of the hind legs. This could be attributed to the fact that the external genitals and inguinal/groin region of the body are highly supplied with blood, thinner and short hair skin. Ticks usually prefer thinner and short hair skin for infestation, as it helps with easy penetration of mouth parts into richly vesicular areas for blood feeding [46]. The higher proportion of ticks in these predilection sites could be due to high supply of blood and the difficulty in reaching them with hand spray which was the method of application for tick control, as reported in all the study areas. Similar findings have been reported in previous studies [24, 40]. Findings from this study were similar and confirm the report of other investigators [24, 39, 47]. In general, most of ticks in this study were observed to infest sites with shorter hair and thinner skin. These sites could facilitate penetration of tick mouth parts and allow better access to the blood circulatory system for feeding [48]. Moreover, the distribution of ixodid ticks in different predilection sites may involve complex intrinsic behaviours that are under chemical control. Different pheromones, which emanate from the anus, coxal glands, and female genital aperture, control other behaviours such as aggregation, clasping and attachment during mating attraction, and potential mate recognition in males, mounting, and copulation [33, 49]. Furthermore, a variety of factors such as host diversity, interaction between tick species, time and season, and inaccessibility of grooming determines the attachment of ticks to the host’s skins [40].

The higher number of males than females observed in *Amblyomma* and *Hyalomma* spp. could be due to their preference for the selected animal body zones, including armpit, groin, udder, and scrotum. This preference leads to clusters with few females, resulting in a concentration on more males than females on the attachment site. The higher number of females than males of *Rhipicephalus* spp. could be due to the difficulty in collecting them from the host animal due to their smaller sizes [13, 34, 36].

In this study, the mitochondrial CO1 and 16S rRNA genes were successfully amplified and these makes the first barcoding sequences of Ixodid ticks reported from Kilombero and Iringa Districts. Similar findings have been reported in the previous studies from Brazil [50], Republic of China [22], Tanzania [29], Uganda [23], and Malaysia [51]. The small sequence differences observed in this study could be due to intraspecific variations and in some cases, it could be due to low sequence quality in few of the samples, especially when nucleotides with weak signal were present in a sequence [22, 52]. The observed low nucleotide sequence identity of 96.08% compared to the respective reference sequence in the GenBank for *H. albiparmatum* in this study most likely reflects the presence of intra-species genetic variation between ticks from the same species adopted to various geographical regions of different countries as described by previous studies [53–55]. Interestingly, the registered sequence data of mitochondrial CO1 barcoding gene of *A. gemma* were not available on GenBank, however it was available on BOLD (www.barcodinglife.org). The lack of corresponding mitochondrial CO1 gene sequences in GenBank for *A. gemma* and intra-species genetic variation could be considered one of the limitations of the molecular approach for tick species identification [55]. The evolutionary and phylogenetic analyses of ticks using DNA barcoding system could be utilized to discriminate ticks within existing classification systems. The traditional taxonomic traits like lifecycle and morphological characters should be considered in addition to one or several genes when a new species or subspecies is to be determined.

## Conclusion

This study reports the abundance and distribution of Ixodid ticks on cattle in Kilombero and Iringa Districts. The morphological and molecular identification of ticks has greatly expanded the understanding of the geographical distribution and phylogenetic relationship of the tick species. Therefore, it is necessary to explore the epidemiological and molecular aspects of various tick species in other regions of Tanzania. This study will be useful in the investigation and designing control strategies for tick control.

## Data Availability

The dataset generated during the current study are available at the NCBI GenBank database (Accession numbers: OM974109, OM974110, OM974111, OM974112, OM978262, OM978264 and OM978265).

## Abbreviations

BOLD: Barcode of Life Database
CO1: Cytochrome c Oxidase subunit 1
rRNA: ribosomal Ribonucleic Acid
SUA: Sokoine University of Agriculture
TBDs: Tick Borne Diseases

## References

[1] Isack IK, Walter M, Sebastian C, Marja K, Seong-Gu H, Martin S (2017). Abundance and distribution of Ixodid tick species infesting cattle reared under traditional farming systems in Tanzania. Afr J Agric Res.

[2] Ćakić S, Mojsilović M, Mihaljica D, Milutinović M, Petrović A, Tomanović S (2014). Molecular characterization of CO1 gene of Ixodes Ricinus (LINNAEUS, 1758) from Serbia. Arch Biol Sci.

[3] Rehman A, Nijhof AM, Sauter-louis C, Schauer B, Staubach C, Conraths FJ. Distribution of ticks infesting ruminants and risk factors associated with high tick prevalence in livestock farms in the semi- arid and arid agro-ecological zones of Pakistan. Parasites & Vectors 2017;1–15. 10.1186/s13071-017-2138-0 PMC5395890 28420420

[4] United Republic of Tanzania (URT) (2011). Livestock Sector Development Programme. Ministry of Livestock and Fisheries Development.

[5] Namgyal J. Identification and Distribution of Tick Species in Cattle in Eastern Bhutan, MSc Thesis, University of Calgary, Calgary, AB. 2020. p. 1–182.

[6] United Republic of Tanzania (URT). Tanzania Livestock Sector Analysis (2016/2017-2031-2032). Volume 53, 4 ed. Ministry of Agriculture, Livestock and Fisheries; 2017. pp. 1–145.

[7] Mamiro KA, Magwisha HB, Rukambile EJ, Ruheta MR, Kimboka EJ, Malulu DJ (2016). Occurrence of ticks in cattle in the New Pastoral Farming Areas in Rufiji District, Tanzania. J Vet Med.

[8] Lynen G, Zeman P, Bakuname C, Di G, Paul G, Paul M et al. Cattle ticks of the genera Rhipicephalus and Amblyomma of economic importance in Tanzania: distribution assessed with GIS based on an extensive field survey. Exp Appl Acarol. 2007;(43):303–19. 10.1007/s10493-007-9123-9 18044004

[9] Kwak YS, Kim TY, Nam S, Lee I, Kim H, Mduma S (2014). Ixodid Tick Infestation in Cattle and Wild Animals in Maswa.

[10] Nchu F, Nyangiwe N, Muhanguzi D, Nzalawahe J, Nagagi YP, Msalya G (2020). Development of a practical framework for sustainable surveillance and control of ticks and tick-borne diseases in Africa. Vet World.

[11] Emmanuel NH, Adrian M (2012). Tick infestations in extensively grazed cattle and efficacy trial of high-cis cypermethrin pour-on preparation for control of ticks in Mvomero district in Tanzania. BioMeC Vet Res.

[12] Balama C, Augustino S, Eriksen S, Makonda FSB, Amanzi N (2013). Climate change adaptation strategies by local farmers in Kilombero District, Tanzania. Ethiop J Environ Stud Manag.

[13] Silatsa BA, Simo G, Githaka N, Mwaura S, Kamga RM, Oumarou F et al. A comprehensive survey of the prevalence and spatial distribution of ticks infesting cattle in different agro-ecological zones of Cameroon. Parasites and Vectors. 2019;12(1):1–14. Available from: 10.1186/s13071-019-3738-7. 10.1186/s13071-019-3738-7 PMC6796472 31623642

[14] Marcellino WL, Julla II, Salih DA, El Hussein ARM (2011). Ticks infesting cattle in Central Equatoria region of South Sudan. Onderstepoort J Vet Res.

[15] Walker AR, Bouattour A, Camicas LJ, Estrada-peña A, Horak IG (2014). Ticks of domestic animals in Africa: a guide to identification of species. Univ Edinb.

[16] Hoskins JD. Keys to their Identification. Vet Clin North Am Small Anim Pract. 1991;21(1):185–97. Available from: 10.1016/S0195-5616(91)50018-8. 10.1016/s0195-5616(91)50018-8 2014622

[17] Mehravaran A, Moradi M, Telmadarraiy Z, Mostafavi E, Reza A. Ticks and Tick-borne Diseases Molecular detection of Crimean-Congo haemorrhagic fever (CCHF) virus in ticks from southeastern Iran. Ticks Tick Borne Dis. 2013;4(1–2):35–8. Available from: 10.1016/j.ttbdis.2012.06.006. 10.1016/j.ttbdis.2012.06.006 23238248

[18] Sherifi K, Rexhepi A, Berxholi K, Mehmedi B, Gecaj RM, Hoxha Z (2018). Crimean-Congo hemorrhagic fever virus and Borrelia burgdorferi sensu lato in ticks from Kosovo and Albania. Front Vet Sci.

[19] Tekin S, Bursali A, Mutluay N, Keskin A, Dundar E (2012). Veterinary Parasitology Crimean-Congo hemorrhagic fever virus in various ixodid tick species from a highly endemic area. Vet Parasitol.

[20] Lv J, Wu S, Zhang Y, Chen Y, Feng C, Yuan X (2014). Assessment of four DNA fragments (COI, 16S rDNA, ITS2, 12S rDNA) for species identification of the Ixodida (Acari : Ixodida). Parasit Vectors.

[21] Lv J, Wu S, Zhang Y, Zhang T, Feng C, Jia G (2014). Development of a DNA barcoding system for the Ixodida (Acari: Ixodida). Mitochondrial DNA.

[22] Chitimia L, Iustin RL, Wu CX (2010). Genetic characterization of ticks from southwestern Romania by sequences of mitochondrial cox 1 and nad 5 genes. Exp Appl Acarol.

[23] Muhanguzi D, Byaruhanga J, Amanyire W, Ndekezi C, Ochwo S, Nkamwesiga J (2020). Invasive cattle ticks in East Africa: morphological and molecular confirmation of the presence of Rhipicephalus microplus in south-eastern Uganda. Parasites and Vectors.

[24] Kemal J, Tamerat N, Tuluka T (2016). Infestation and identification of Ixodid Tick in cattle: the case of Arbegona District, Southern Ethiopia. J Vet Med.

[25] Kumar S, Stecher G, Li M, Knyaz C, Tamura K (2018). MEGA X: molecular evolutionary genetics analysis across computing platforms. Mol Biol Evol.

[26] Damian D, Damas M, Wensman JJ, Berg M (2021). Molecular diversity of hard tick species from selected areas of a wildlife-livestock interface ecosystem at mikumi national park, Morogoro Region, Tanzania. Vet Sci.

[27] Swai ES, Karimuribo ED, Rugaimukamu EA, Kambarage DM (2006). Factors influencing the distribution of questing ticks and the prevalence stimation of T. parva infection in brown ear ticks in the Tanga region, Tanzania. J Vector Ecol.

[28] Mapholi NO, Marufu MC, Maiwashe A, Banga CB, Muchenje V, Macneil MD et al. Towards a genomics approach to tick (Acari: Ixodidae) control in cattle. Article in press. Ticks Tick Borne Dis. 2014;1–9. 10.1016/j.ttbdis.2014.04.006 24954600

[29] Copland J, Floyd R, Gibson J, Hibberd S, Sutherst R, Thorne L (1986). Ticks and Tick-borne Diseases Proceedings of an international workshop on the ecology of ticks and epidemiology of tick-borne diseases, held at Nyanga Ministry of Lands, Agriculture and rural settlement, Zimbabwe. ACIAR Proc.

[30] Kaiser MN, Bourne AS (1977). Relationship between ticks and zebu cattle in southern uganda. Control.

[31] Tatchell RJ, Easton E (1986). Tick (Acari: Ixodidae) ecological studies in Tanzania. Bull Entomol Res.

[32] Okello-Onen J, Tukahirwa EM, Perry BD, Rowlands GJ, Nagda SM, Musisi G (1999). Population dynamics of ticks on indigenous cattle in a pastoral dry to semi-arid rangeland zone of Uganda. Exp Appl Acarol.

[33] Lorusso V, Picozzi K, De Bronsvoort BMC, Majekodunmi A, Dongkum C, Balak G (2013). Ixodid ticks of traditionally managed cattle in central Nigeria: where Rhipicephalus (Boophilus) microplus does not dare (yet?). Parasites and Vectors.

[34] Swai ES, French NP, Karimuribo ED, Fitzpatrick JL, Bryant MJ, Brown PE (2005). Spatial and management factors associated with exposure of smallholder dairy cattle in Tanzania to tick-borne pathogens. Int J Parasitol.

[35] Hughes VL, Randolph SE (2001). Testosterone increases the transmission potential of tick-borne parasites. Parasitology.

[36] Asrate S, Yalew A (2019). Prevalence of cattle tick infestation in and around Haramaya district, Eastern Ethiopia. Int J Vet Med Anim Heal.

[37] Wasihun P, Doda D. Study on prevalence and identification of ticks in Humbo district, Southern Nations, Nationalities, and People’s Region (SNNPR), Ethiopia. J Vet Med Anim Heal. 2013;5(3):73–80. Available from: http://www.academicjournals.org/JVMAH.

[38] Kerario II, Simuunza M, Laisser ELK, Chenyambuga S. Exploring knowledge and management practices on ticks and tick-borne diseases among agro-pastoral communities in Southern Highlands,. 2018;11:48–57. 10.14202/vetworld.2018.48-57 PMC5813511 29479157

[39] Kazwala RR, Kambarage DM, Daborn CJ, Nyange J, Jiwa SFH, Sharp JM (2001). Risk factors associated with the occurrence of bovine tuberculosis in cattle in the Southern Highlands of Tanzania. Vet Res Commun.

[40] Mazet JAK, Clifford DL, Coppolillo PB, Deolalikar AB, Erickson JD, Kazwala RR (2009). A “One health” approach to address emerging zoonoses: the HALI project in Tanzania. PLoS Med.

[41] Roug A, Muse EA, Clifford DL, Paul G, Mpanduji D, Makingi G (2020). Health of african buffalos (Syncerus caffer) in Ruaha national park, tanzania. J Wildl Dis.

[42] Wang’ang’a Oundo J, Villinger J, Jeneby M, Ong’amo G, Otiende MY, Makhulu EE et al. Pathogens, endosymbionts, and blood-meal sources of host-seeking ticks in the fast-changing Maasai Mara wildlife ecosystem. PLoS One. 2020;15(8):1–16. Available from: 10.1371/journal.pone.0228366. 10.1371/journal.pone.0228366 PMC7458302 32866142

[43] Asmaa NM, ElBably MA, Shokier KA. Studies on prevalence, risk indicators and control options for tick infestation in ruminants. Beni-Suef Univ J Basic Appl Sci. 2014;3(1):68–73. Available from: 10.1016/j.bjbas.2014.02.009.

[44] Gudina E, Gatachew Y, Asebe G (2016). Distribution and prevalence of Hard Tick in cattle and around. Int J Agric Earth Sci.

[45] Sajid MS, Iqbal Z, Khan MN, Muhammad G, Khan MK (2009). Prevalence and associated risk factors for bovine tick infestation in two districts of lower Punjab, Pakistan. Prev Vet Med.

[46] Ikpeze OO, Eneanya CI, Chinweoke OJ, Aribodor DN, Anyasodor AE. Species diversity, distribution and predilection sites of ticks (Acarina: ixodidae) on trade cattle at Enugu and Anambra States, south-eastern Nigeria. Zool. 2011;9:1–8. Available from: http://ajol.info/index.php/tzool/article/view/73121.

[47] Caetano RL, Vizzoni VF, Bitencourth K, Sato TP, Pinto ZT, Vilges S (2017). Morphology, Systematics, evolution ultrastructural morphology and molecular analyses of Tropical and Temperate “ Species ” of Rhipicephalus sanguineus sensu lato (Acari : Ixodidae) in Brazil. J of Medical Entomol.

[48] Ernieenor FCL, Apanaskevich DA, Ernna G, Mariana A. Description and characterization of questing hard tick, Dermacentor steini (Acari: Ixodidae) in Malaysia based on phenotypic and genotypic traits. Exp Appl Acarol. 2020;80(1):137–49. Available from: 10.1007/s10493-019-00439-4. 10.1007/s10493-019-00439-4 31832837

[49] Gou H, Xue H, Yin H, Luo J, Sun X. Molecular characterization of hard ticks by cytochrome c oxidase subunit 1 sequences. 2018;56(6):583–8. 10.3347/kjp.2018.56.6.583 PMC6327197 30630279

[50] Beati L, Keirans JE (2001). Analysis of the systematic Relationships among ticks of the Genera Rhipicephalus and Boophilus (Acari: Ixodidae) based on mitochondrial 12S ribosomal DNA gene sequences and morphological characters. J Parasitol.

[51] Dantas-Torres F, Otranto D (2013). Species diversity and abundance of ticks in three habitats in southern Italy. Ticks Tick Borne Dis [Internet].

[52] Kumsa B, Laroche M, Almeras L, Mediannikov O, Raoult D, Parola P (2016). Morphological, molecular and MALDI-TOF mass spectrometry identification of ixodid tick species collected in Oromia, Ethiopia. Parasitol Res.

[53] Saitou N, Nei M (1987). The neighbor-joining method: a New Method for reconstructing phylogenetic trees. Mol Biol Evol.

[54] Felsenstein J (1985). Confidence limits on phylogenies: an approach using the bootstrap. Evol (N Y).

[55] Tamura K, Nei M, Kumar S (2004). Prospects for inferring very large phylogenies by using the neighbor-joining method. Proc Natl Acad Sci U S A.

